# Supplementary material for: Multiple configurations and fluctuating trophic control in the Barents Sea food-web
Source: PLoS One. 2021 Jul 9;16(7):e0254015. doi: 10.1371/journal.pone.0254015 (PMC8270156; doi:10.1371/journal.pone.0254015)
Supplement: S1 File — (DOCX) [file pone.0254015.s001.docx]

# Supplementary materials

## Mathematical formulation of the Non-Deterministic Network Dynamic model (NDND)

There are five biological and physical constraints in the NDND model: satiation (maximum ingestion rate), inertia (maximum growth rate and maximum death rate), positive flows (all flow values are equal or greater than 0) and refuge biomass (biomass threshold under which the prey is not available for predators) [1].

The mathematical formulation of the constraints is as follows:

(1) the food intake is limited by satiation (*σ*),

| $\sum_{j} F_{ji}\leq\sigma_{i}B_{i}$ | *(1.1)* |
| --- | --- |

(2) variations of biomass for each species is bounded by a maximum growth rate (*ρ*),

| $B_{i,t+1}\leq e^{\rho_{i}}B_{i,t}+ {\frac{1-e^{-\mu_{i}}}{\mu_{i}}I}_{i}$ | *(1.2)* |
| --- | --- |

(3) and by a maximal mortality rate (*-ρ*),

| $e^{{-\rho}_{i}}B_{i,t}\leq B_{i,t+1}$ | *(1.3)* |
| --- | --- |

(4) the flows between species must be positive:

| $F_{ij}\geq0$ | *(1.4)* |
| --- | --- |

(5) species are not available for predation if their biomass is below a certain value (*β*),

| $B_{i}\geq\beta_{i}$ | *(1.5)* |
| --- | --- |

The biological and physical constraints define a polytope. It represents all combinations of trophic flows of the food-web topology which respect the constraints we have defined above. The polytope is sampled uniformly using the Complex Polytope Gibbs Sampler (cpgs) from the RCaN package [2] to obtain one combination of possible flows [3,4]. The sampled flow combination is then used to estimate the biomass of the species at the next time step using the equation 1.6.

| $B_{i,t+1}=e^{-\left( \mu_{i} \right)}B_{i,t}+\frac{1-e^{-\left( \mu_{i} \right)}}{\mu_{i}}\left[ \gamma_{i}\sum_{j} F_{ji}ϗ_{i}+I_{i}-\sum_{j} F_{ij}-E_{i} \right]$ | *(1.6)* |
| --- | --- |

*µ_i_* is the parameter for metabolic losses of species *i*, *B_i,t_* and *B_i,t+1_* are the biomass of the species *i* at the time step *t* and t+1, respectively, *γ_i_* is the assimilation efficiency parameter of species *i*, *κ_i_* is the digestibility parameter of species *i*, *F_ji_* is the incoming flow of biomass for species *i*, *F_ij_* is the outcoming biomass flow for species *i*, *I_i_* is the import biomass of species *i* and *E_i_* is the export biomass of species.

## Summary of performed statistical analysis.


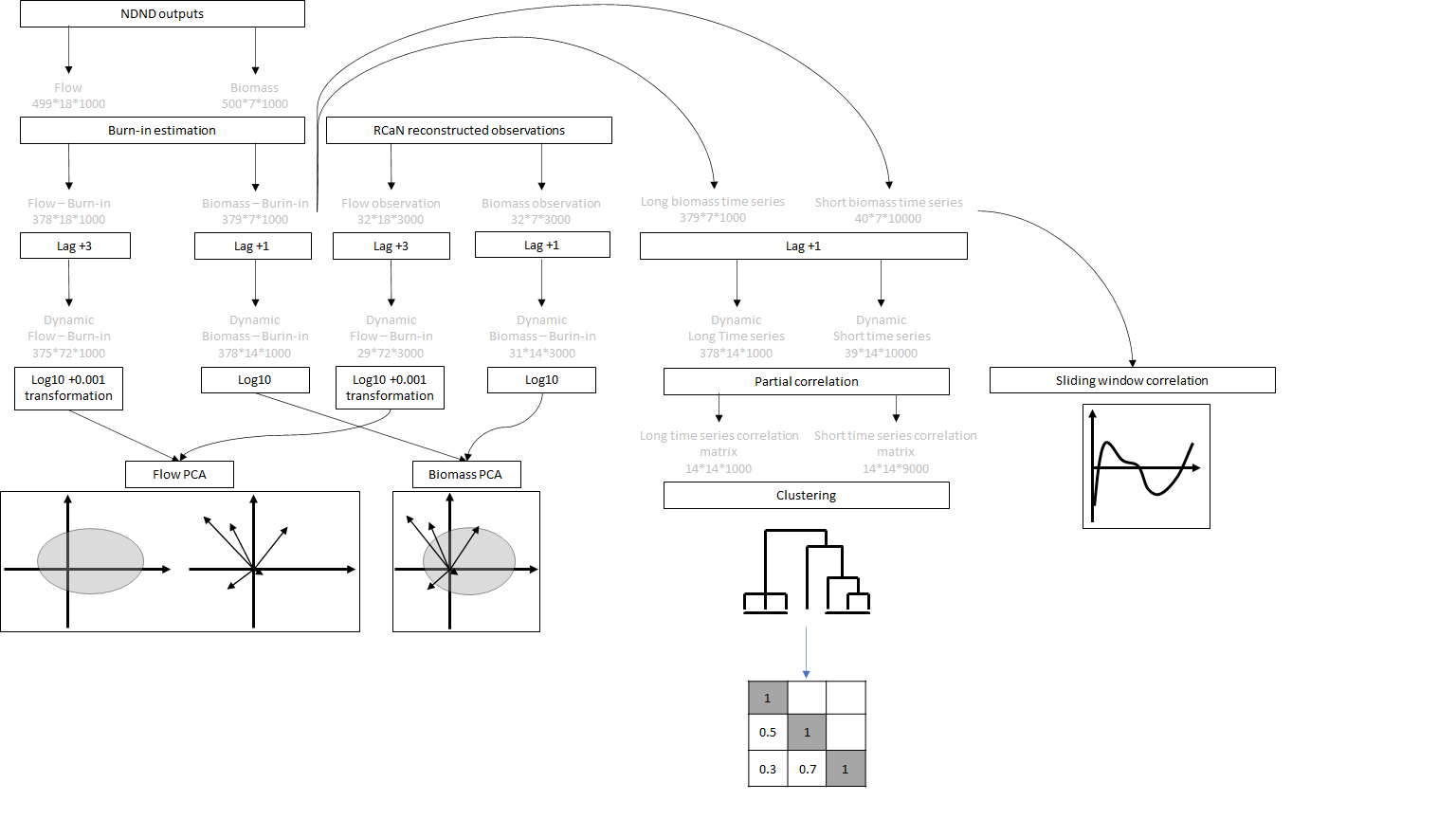


Figure 1 Flow chart summarizing the statistical analysis performed in the study. Elements in grey correspond the dimensions of the matrix used at each step. Boxes indicate the different steps of the analysis (data manipulation or analysis).

## Method for burn-in period estimation

To ensure that we perform the statistical analysis on data which is independent from input biomass, we estimate a burn-in period based on an autocorrelation analysis. We estimate the temporal autocorrelation of each time-series (Figure 2), and define the significance threshold as:

| $\pm\frac{{z_{1-\alpha}}/2}{\sqrt{N}}$ | *(3.1)* |
| --- | --- |

Where, z is the cumulative distribution of the standard normal distribution, α is the significance level (here set to 0.975), and N is the number of years remaining in the simulation (e.g. if we estimate the correlation between the biomass at year 20 and year 1, then N equals 480). The measured correlation is considered non-significant if it is lower than the significance level.

The first year for which the estimated correlation is not significant is retained for each run and each species. A median year is estimated across all runs for each species and the highest median year is retained as the length of the burn-in period (black dashed line in figure 2).


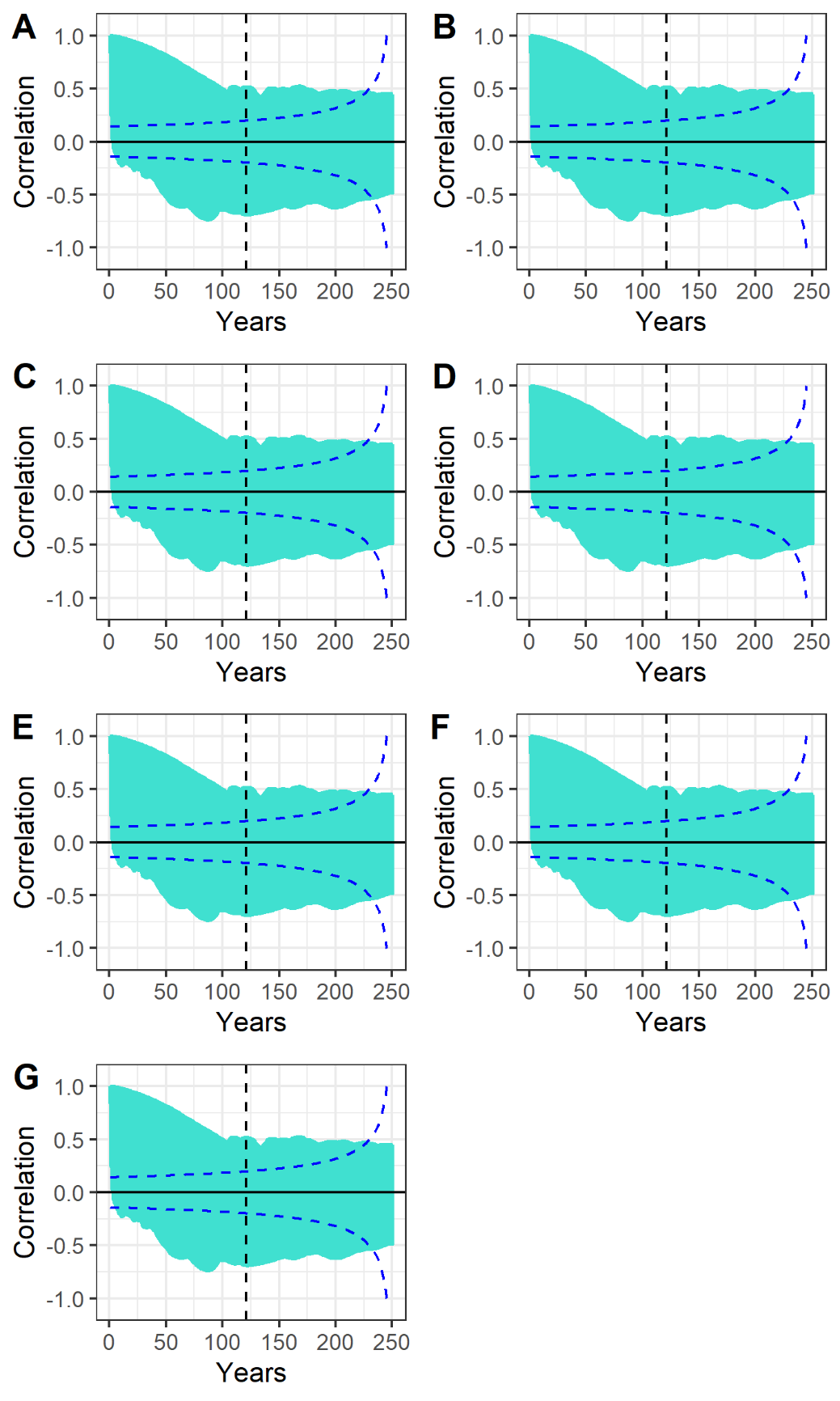


Figure 2 Autocorrelation plot for herbivorous zooplankton, omnivorous zooplankton, benthos, pelagic fish, demersal fish, marine mammals, and birds. Each trajectory corresponds to a run. Blue dotted lines are the significance threshold, black dotted line correspond to the estimated burn-in period.

## Setup of the CaN model for the Barents Sea

The parametrization of the CaN model to reconstruct observation time series is taken from [4]. Parameter values were revised to match the parameter values of the NDND simulations. In the CaN model, fisheries are considered for omnivorous zooplankton, pelagic fish, demersal fish and mammals. Time series of biomass, consumption by cod and landings used to constrain the CaN model are taken from [5]. Marine mammal biomass time-series were taken from the NAMMCO group monitoring data (personal communication). Primary production estimates for the time-period 1998-2018 were derived from satellite measures of Chlorophyll-a from [5]. Table 4.1 presents the parameter values of the CaN model used in this study and Table 4.2 present the historical biomass time-series used to constrain the CaN model. Historical landings time-series are presented in Table 4.3. Estimations of prey consumption by cod are presented in Table 4.4.

Table 4.1 CaN model parameters for the seven species of the simplified Barents Sea food-web.

| Group | Primary production | Herbivorous zooplankton | Omnivorous zooplankton | Benthos | Pelagic | Demersal | Mammals | Birds |
| --- | --- | --- | --- | --- | --- | --- | --- | --- |
| Assimilation (γ) |  | 1 | 1 | 0.94 | 0.9 | 0.93 | 1 | 0.84 |
| Digestibility (κ) | 0.65 | 0.9 | 0.9 | 0.6 | 0.9 | 0.85 |  |  |
| Losses (µ) |  | 8.4 | 5.5 | 1.5 | 2.85 | 1.65 | 5.5 | 74 |
| Satiation (σ) |  | 128 | 42 | 25.2 | 13.5 | 5.5 | 10.9 | 123 |
| Inertia (ρ) |  | 3.8 | 1.77 | 0.23 | 1.3 | 0.18 | 0.06 | 0.27 |
| Uncertainty on bounds (+) (υ) | 1.3 | 2.5 | 1.6 | 1.5 | 1.25 | 1.25 | 1.5 | 2 |
| Uncertainty on bounds (-) (λ) | 0.7 | 0.4 | 0.6 | 0.6 | 0.8 | 0.8 | 0.6 | 0.5 |

Table 4.2 Biomass time-series for estimated primary production, herbivorous zooplankton, omnivorous zooplankton, benthos, pelagic fish, demersal fish, mammals and birds used to constrain the reconstructions of Barents Sea food-web trajectories using the RCaN model. Values are given in thousand tons.

| Year | Primary production | Herbivorous zooplankton | Omnivorous zooplankton | Benthos | Pelagic | Demersal | Mammals | Birds |
| --- | --- | --- | --- | --- | --- | --- | --- | --- |
| 1988 | 1000000 | 25432,1229 | 24275,6145 | 105000 | 428,277 | 1339,32 | 560 | 11 |
| 1989 | 1000000 | 31987,2 | 16130,85 | 105000 | 864,5218 | 1290,936 | 560 | 11 |
| 1990 | 1000000 | 23027,7261 | 7481,53736 | 105000 | 5831,6563 | 1294,721 | 560 | 11 |
| 1991 | 1000000 | 21188,3366 | 16833,3622 | 105000 | 7288,5585 | 1823,767 | 560 | 11 |
| 1992 | 1000000 | 27314,0224 | 7940,31072 | 105000 | 5152,5039 | 2257,915 | 560 | 11 |
| 1993 | 1000000 | 37612,3072 | 11880,4064 | 105000 | 799,6427 | 2946,711 | 560 | 11 |
| 1994 | 1000000 | 72438,1894 | 22699,6218 | 105000 | 203,9375 | 2886,881 | 560 | 11 |
| 1995 | 1000000 | 57941,7824 | 23526,6004 | 105000 | 195,6632 | 2628,441 | 560 | 11 |
| 1996 | 1000000 | 38465,0374 | 24633,2476 | 105000 | 504,2064 | 2460,214 | 560 | 11 |
| 1997 | 1000000 | 43364,7488 | 19153,7093 | 105000 | 912,1547 | 2117,867 | 560 | 11 |
| 1998 | 799240,58 | 39682,56 | 20505,952 | 105000 | 2057,4555 | 1833,737 | 560 | 11 |
| 1999 | 747073,95 | 36009,6198 | 15105,0644 | 105000 | 2779,2079 | 1716,531 | 560 | 11 |
| 2000 | 1050000 | 41920,3763 | 14611,7718 | 105000 | 4276,7688 | 1781,169 | 560 | 11 |
| 2001 | 1170000 | 31697,8842 | 16117,2306 | 105000 | 3634,1086 | 2132,369 | 560 | 11 |
| 2002 | 1110000 | 35601,6 | 9402,57 | 105000 | 2212,4722 | 2490,085 | 560 | 11 |
| 2003 | 874724,94 | 31731,2 | 14754,0347 | 105000 | 535,5282 | 2649,912 | 560 | 11 |
| 2004 | 1070000 | 36542,4512 | 24127,2668 | 105000 | 960,2248 | 2616,288 | 560 | 11 |
| 2005 | 909010,84 | 42440,3392 | 21917,7839 | 105000 | 574,6143 | 2661,293 | 560 | 11 |
| 2006 | 1100000 | 45739,1251 | 25576,6826 | 105000 | 1027,101 | 2547,142 | 560 | 11 |
| 2007 | 946826,75 | 39293,3146 | 21387,1126 | 105000 | 2095,59 | 3133,446 | 560 | 11 |
| 2008 | 872083,75 | 35251,2659 | 34031,009 | 105000 | 4466,533 | 4108,277 | 560 | 11 |
| 2009 | 931335,47 | 36147,9002 | 21172,0225 | 105000 | 3822,85 | 4979,174 | 560 | 11 |
| 2010 | 1130000 | 37488,7519 | 28642,7589 | 105000 | 3535,229 | 5467,148 | 560 | 11 |
| 2011 | 1230000 | 31948,2136 | 37204,7369 | 105000 | 3733,877 | 5499,471 | 560 | 11 |
| 2012 | 1110000 | 41283,1248 | 23060,7775 | 105000 | 3708,622 | 5515,659 | 560 | 11 |
| 2013 | 1190000 | 29734,9009 | 24774,2993 | 105000 | 4041,22 | 5608,052 | 560 | 11 |
| 2014 | 1000000 | 39302,4 | 12748,03 | 105000 | 2005,451 | 5201,286 | 560 | 11 |
| 2015 | 1187500 | 50293,1344 | 28425,8662 | 105000 | 1002,389 | 4858,691 | 560 | 11 |
| 2016 | 1500000 | 44224 | 27254,4245 | 105000 | 518,23943 | 4327,74 | 560 | 11 |
| 2017 | 1075000 | 36544 | 29555,63 | 105000 | 2622,433 | 4135,313 | 560 | 11 |
| 2018 | 1362500 | 41024 | 5317,82 | 105000 | 1638,377 | 3699,495 | 560 | 11 |
| 2019 | 1000000 | 45184 | 6080 | 105000 | 455,616 | 3694,9297 | 560 | 11 |

Table 4.3 historical Landings time-series for krill (omnivorous zooplankton), pelagic fish, demersal fish and marine mammals used to constraint the reconstructed flows of fisheries in the Barents Sea. Values are given in thousand tons.

| Year | Omnivorous zooplankton | Pelagic | Demersal | Mammals |
| --- | --- | --- | --- | --- |
| 1988 | 48,689 | 0 | 789,181 | 0,145 |
| 1989 | 62,748 | 0,2 | 538,298 | 0,085 |
| 1990 | 81,164 | 0,1 | 332,848 | 0,025 |
| 1991 | 74,862 | 929 | 454,485 | 0 |
| 1992 | 68,568 | 1143,6 | 703,838 | 1,31845 |
| 1993 | 56,312 | 637,5 | 808,514 | 2,3787 |
| 1994 | 28,287 | 6,1 | 1038,036 | 2,61 |
| 1995 | 25,22 | 23,3 | 1038,377 | 1,8623 |
| 1996 | 34,512 | 20,8 | 1073,576 | 2,73165 |
| 1997 | 35,736 | 7,8 | 1116,032 | 3,1606 |
| 1998 | 55,7901 | 4,5 | 875,951 | 2,9621 |
| 1999 | 75,6692 | 122,7 | 713,285 | 2,79655 |
| 2000 | 83,1703 | 449,6 | 612,796 | 2,7656 |
| 2001 | 57,536 | 609,2 | 647,324 | 3,3355 |
| 2002 | 61,488 | 688,5 | 785,977 | 3,28555 |
| 2003 | 39,225 | 321,3 | 819,282 | 3,57325 |
| 2004 | 42,734 | 1,6 | 895,583 | 2,63995 |
| 2005 | 42,618 | 23,4 | 937,859 | 4,5791 |
| 2006 | 29,627 | 16,3 | 881,905 | 4,21645 |
| 2007 | 29,931 | 30,9 | 832,822 | 3,87165 |
| 2008 | 28,188 | 20,2 | 798,787 | 2,53 |
| 2009 | 27,272 | 324 | 885,356 | 2,425 |
| 2010 | 25,198 | 350,4 | 1054,737 | 2,353 |
| 2011 | 30,226 | 379,5 | 1186,663 | 2,695 |
| 2012 | 24,756 | 296,1 | 1204,25 | 2,3206 |
| 2013 | 19,249 | 177,01 | 1291,582 | 2,97 |
| 2014 | 20,964 | 66 | 1296,041 | 3,68 |
| 2015 | 34,022 | 115 | 1191,415 | 3,3 |
| 2016 | 29,609 | 0 | 1224,606 | 2,95935 |
| 2017 | 29,753 | 0 | 1241,683 | 2,16015 |
| 2018 | 55,911 | 195 | 1151,183 | 2,603 |
| 2019 | 78 | 0 | 1031,191 | 2,2353 |

Table 4.4 Time-series of estimated prey consumption by cod in the Barents Sea. Considered preys are amphipods, krill, shrimp, capelin, herring, polar cod, cod, haddock, redfish, Greenland halibut and blue whiting. Values are given in thousand tons.

| Year | Sp -> Cod | Amphipod -> cod | Krill->Cod | Shrimp->Cod | Capelin->Cod | Herring->Cod | Polarcod->Cod | Cod->Cod | Haddock->Cod | Redfish->Cod | Greenland halibut->Cod | Blue Whiting->Cod |
| --- | --- | --- | --- | --- | --- | --- | --- | --- | --- | --- | --- | --- |
| 1988 | 399,30 | 1230,14 | 300,54 | 126,55 | 337,25 | 8,43 | 91,72 | 8,96 | 2,62 | 222,29 | 0,00 | 4,38 |
| 1989 | 649,72 | 793,22 | 237,33 | 130,82 | 570,11 | 2,92 | 32,05 | 7,64 | 10,29 | 227,29 | 0,00 | 0,00 |
| 1990 | 1341,23 | 137,38 | 84,56 | 195,38 | 1608,16 | 7,12 | 5,92 | 19,30 | 15,48 | 243,44 | 0,00 | 86,88 |
| 1991 | 760,60 | 65,63 | 75,93 | 187,85 | 2890,42 | 8,26 | 11,62 | 26,12 | 20,20 | 311,68 | 7,89 | 9,89 |
| 1992 | 893,05 | 97,96 | 151,31 | 370,22 | 2449,64 | 330,33 | 95,84 | 54,41 | 106,03 | 187,37 | 21,50 | 1,69 |
| 1993 | 737,18 | 247,38 | 671,09 | 312,84 | 3025,47 | 161,91 | 276,37 | 283,11 | 70,25 | 99,93 | 1,96 | 2,24 |
| 1994 | 611,21 | 548,21 | 692,11 | 500,73 | 1082,41 | 145,30 | 566,39 | 214,50 | 47,59 | 77,63 | 0,04 | 0,66 |
| 1995 | 810,76 | 951,38 | 499,72 | 350,71 | 606,07 | 113,18 | 242,21 | 357,81 | 111,36 | 189,25 | 1,45 | 0,39 |
| 1996 | 588,38 | 623,02 | 1138,39 | 334,48 | 529,94 | 46,08 | 101,40 | 520,20 | 66,69 | 96,29 | 0,30 | 9,85 |
| 1997 | 427,43 | 371,91 | 502,40 | 301,96 | 875,09 | 5,39 | 111,42 | 331,15 | 39,83 | 35,55 | 0,00 | 32,15 |
| 1998 | 401,31 | 347,85 | 448,21 | 313,74 | 690,30 | 82,22 | 143,57 | 151,87 | 31,23 | 8,83 | 0,00 | 13,22 |
| 1999 | 376,76 | 142,63 | 270,75 | 246,41 | 1696,60 | 126,50 | 217,21 | 61,21 | 25,55 | 15,71 | 1,01 | 30,54 |
| 2000 | 379,63 | 164,18 | 451,51 | 443,74 | 1706,36 | 52,65 | 190,63 | 75,06 | 50,19 | 8,02 | 0,03 | 37,23 |
| 2001 | 692,75 | 172,00 | 374,66 | 278,05 | 1738,83 | 71,80 | 253,14 | 69,39 | 49,66 | 6,14 | 0,75 | 153,79 |
| 2002 | 379,95 | 95,16 | 254,36 | 241,27 | 2008,02 | 87,39 | 283,98 | 109,35 | 129,09 | 0,63 | 0,09 | 240,32 |
| 2003 | 562,88 | 292,65 | 546,11 | 240,18 | 2188,20 | 216,46 | 283,60 | 115,37 | 173,58 | 3,41 | 0,00 | 78,11 |
| 2004 | 631,35 | 563,79 | 343,54 | 245,79 | 1260,61 | 211,96 | 355,35 | 128,36 | 200,78 | 3,20 | 11,75 | 57,97 |
| 2005 | 776,14 | 575,86 | 522,03 | 268,33 | 1377,59 | 129,98 | 384,18 | 118,02 | 319,68 | 2,35 | 4,73 | 115,58 |
| 2006 | 898,12 | 227,88 | 1098,70 | 368,03 | 1770,79 | 168,90 | 111,17 | 80,55 | 365,06 | 11,90 | 1,68 | 162,20 |
| 2007 | 1355,78 | 325,45 | 1182,78 | 475,25 | 2325,50 | 290,95 | 280,66 | 88,78 | 391,67 | 51,54 | 0,31 | 44,10 |
| 2008 | 1758,66 | 182,14 | 1038,47 | 441,71 | 3213,35 | 114,80 | 560,04 | 207,77 | 311,97 | 67,93 | 13,15 | 18,59 |
| 2009 | 1717,51 | 279,28 | 688,45 | 309,07 | 4602,51 | 138,17 | 842,74 | 226,96 | 289,20 | 33,56 | 2,91 | 5,86 |
| 2010 | 1858,01 | 479,66 | 1154,61 | 330,30 | 4533,06 | 60,86 | 380,46 | 283,31 | 306,35 | 163,52 | 11,88 | 16,78 |
| 2011 | 1785,64 | 287,55 | 986,03 | 256,35 | 4735,61 | 94,65 | 487,78 | 330,35 | 322,97 | 132,80 | 0,25 | 29,74 |
| 2012 | 2309,02 | 346,97 | 875,40 | 397,48 | 4242,26 | 55,65 | 616,92 | 436,44 | 259,47 | 59,03 | 40,64 | 9,92 |
| 2013 | 1971,53 | 278,45 | 570,06 | 292,29 | 4113,49 | 57,02 | 158,46 | 439,47 | 236,41 | 132,89 | 1,36 | 24,55 |
| 2014 | 1546,02 | 324,90 | 472,91 | 211,71 | 4043,07 | 75,86 | 34,92 | 392,94 | 99,27 | 34,59 | 12,84 | 20,44 |
| 2015 | 1672,67 | 637,73 | 629,19 | 240,32 | 3391,02 | 130,55 | 159,40 | 229,20 | 186,90 | 140,82 | 50,55 | 59,42 |
| 2016 | 1691,31 | 525,13 | 717,32 | 294,65 | 2228,26 | 99,27 | 356,55 | 209,57 | 220,03 | 57,24 | 6,42 | 87,63 |
| 2017 | 994,62 | 118,74 | 543,50 | 231,47 | 2808,53 | 175,16 | 86,37 | 309,99 | 263,13 | 41,97 | 5,15 | 23,00 |
| 2018 | 929,90 | 245,91 | 601,15 | 160,93 | 2621,16 | 179,78 | 241,36 | 216,57 | 250,21 | 32,50 | 69,74 | 44,82 |
| 2019 | 759,35 | 168,14 | 483,88 | 303,66 | 2412,09 | 166,28 | 168,40 | 182,62 | 199,34 | 45,71 | 0,00 | 3,72 |

Using the CaN model, we reconstructed biomass time-series for herbivorous zooplankton, omnivorous zooplankton, benthos, pelagic fish, demersal fish, marine mammals and birds. Trophospecies for which data was not available (i.e. benthos, marine mammals and birds) display more variability and larger envelopes than the other trophospecies (Figure 3). For herbivorous zooplankton and omnivorous zooplankton, time series of biomass were used to constrain the reconstructions of biomass time-series. However, there is little confidence in this data because following the dynamics of these species is a challenge. In the CaN model, to integrate the lower confidence in the data, we have implemented larger uncertainties for herbivorous and omnivorous zooplankton than for pelagic and demersal fish. The reconstructed biomass time-series of pelagic and demersal fish are more constrained than the reconstructed time-series of all other trophospecies. It reflects that at an ecosystem level, there is more information on these two trophic groups than for all the other groups. The reconstructed time-series we present here were used to compare the simulated food-web configurations with the food-web configurations of the last three decades.


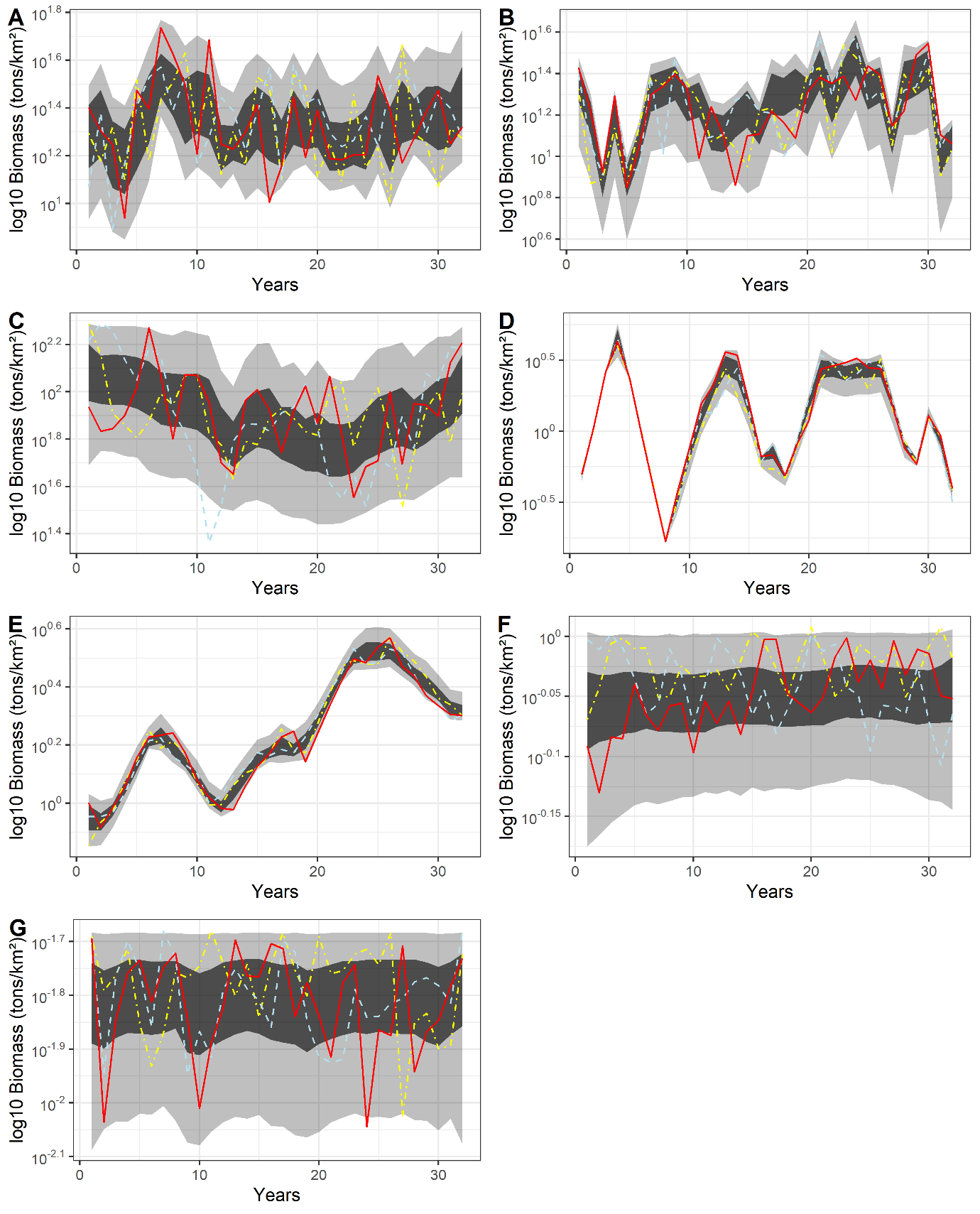


Figure 3 Reconstructed time-series of biomass for herbivorous zooplankton (A), omnivorous zooplankton (B), benthos (C), pelagic fish (D), demersal fish (E), marine mammals (F), and birds (G) for the period 1988-2019. Represented biomass is on log10 scale. The dark grey and light grey envelopes include 50% and 95% of reconstructed biomass, respectively. The colored lines (red-plain, yellow-dash-dotted, and light blue-dashed) represent three randomly selected simulations.

## Estimate of the needed lag to account for temporal autocorrelation.

To perform the dynamical Principal Component Analysis (dPCA), we need to estimate the lag (i.e. time step at which the values is not dependent on the initial input value). The lag is estimated based on the partial temporal autocorrelation estimated for the biomass and the flows of each trophospecies. The partial temporal autocorrelation of biomass indicated that a lag of 1 years is sufficient to ensure that there is no correlation with the input biomass (Figure 4). The flows partial autocorrelation analysis displayed autocorrelation for longer lags (Figure 5). For simulated flow data, we set the lag to 3 years.


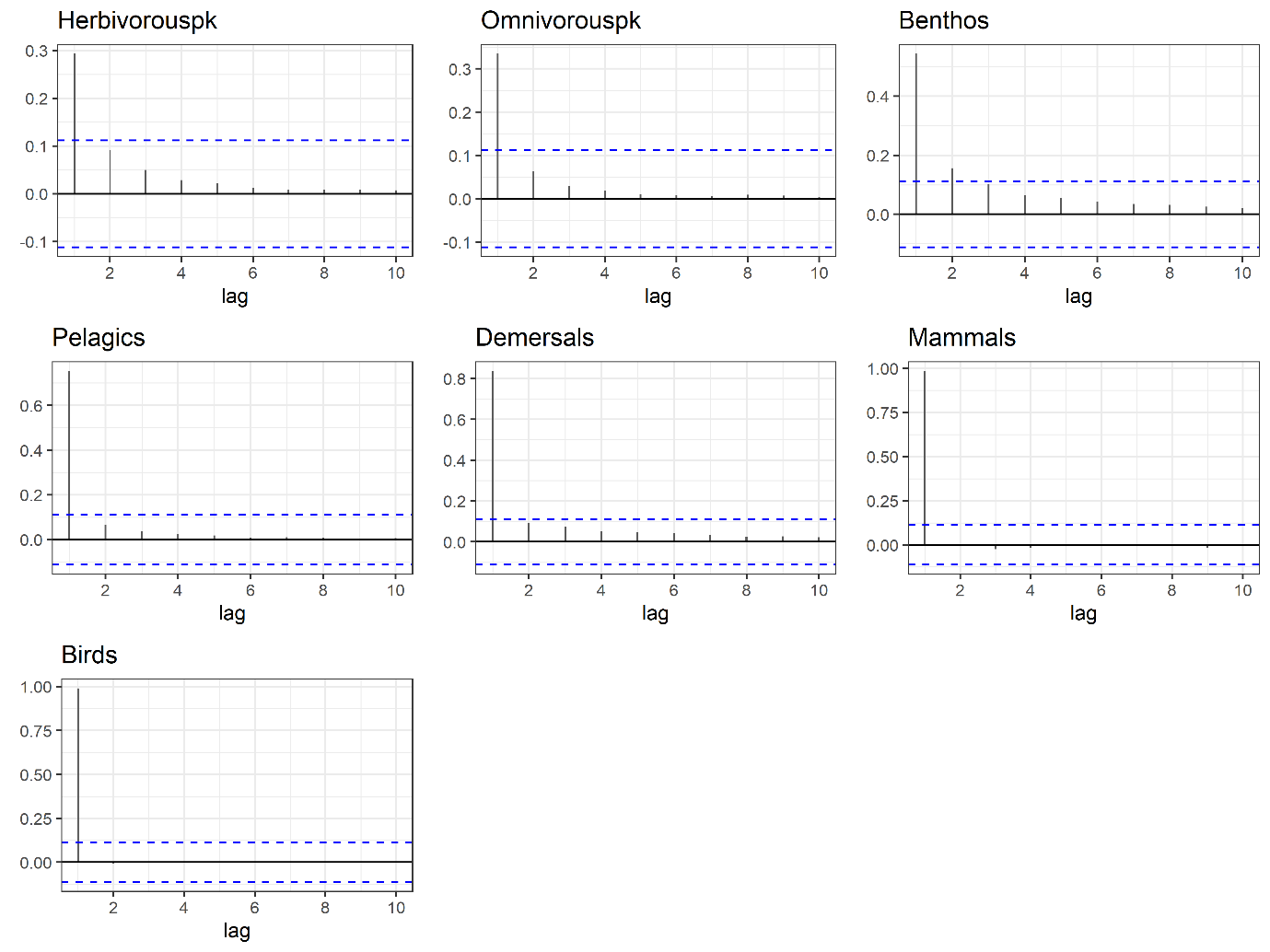


Figure 4 Partial autocorrelograms of simulated biomass time-series. Blue dotted lines correspond to the significance level for 414 samples.


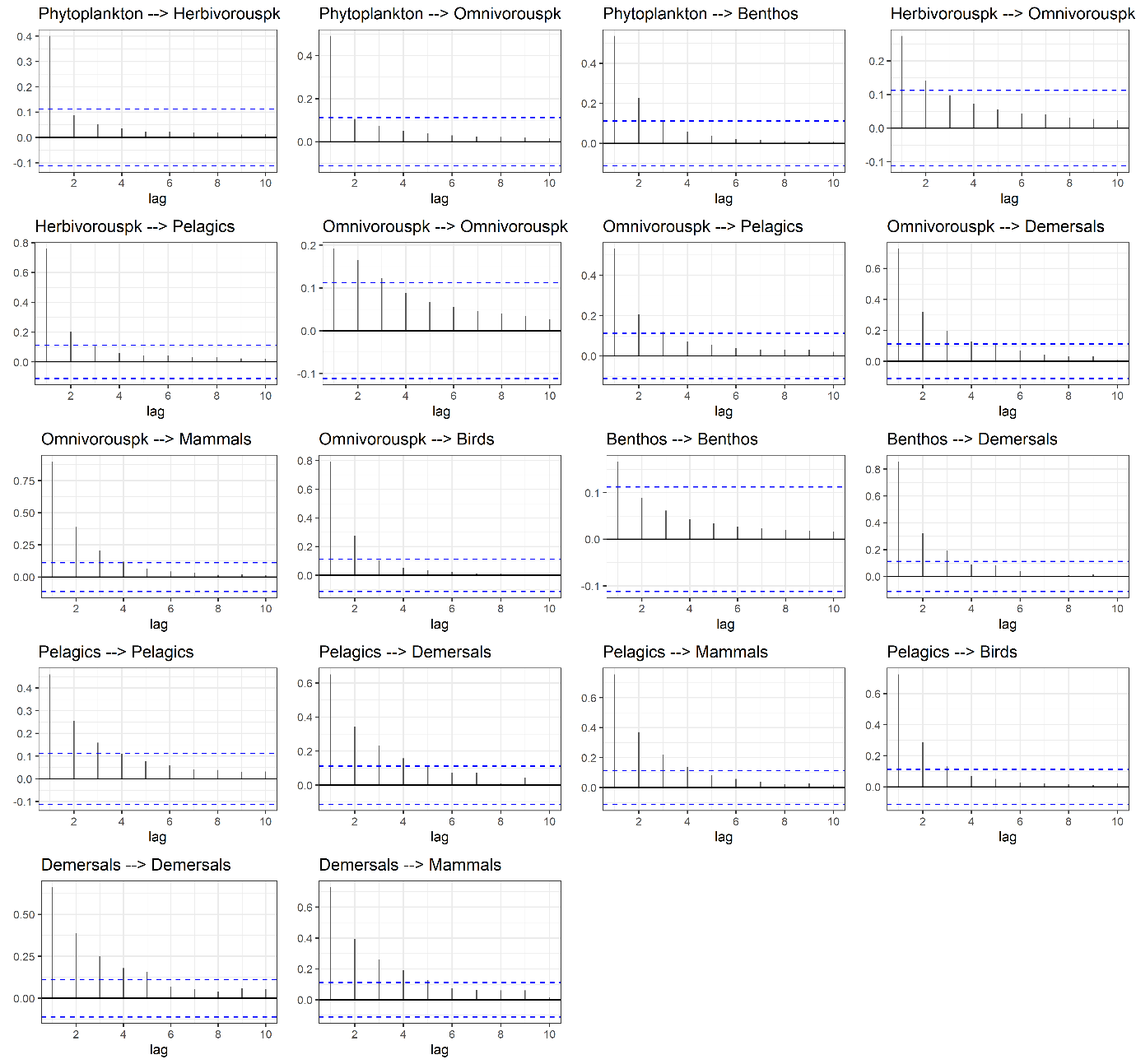


Figure 5 Partial autocorrelograms of simulated flow time-series. Blue dotted lines correspond to the significance level for 413 samples.

## Clustering of partial correlation matrices

The dendrogram of long time-series (379 years) displayed 3 clusters (Figure 6), whereas the dendrogram of short time-series (40 years) displayed four clusters (Figure 6).


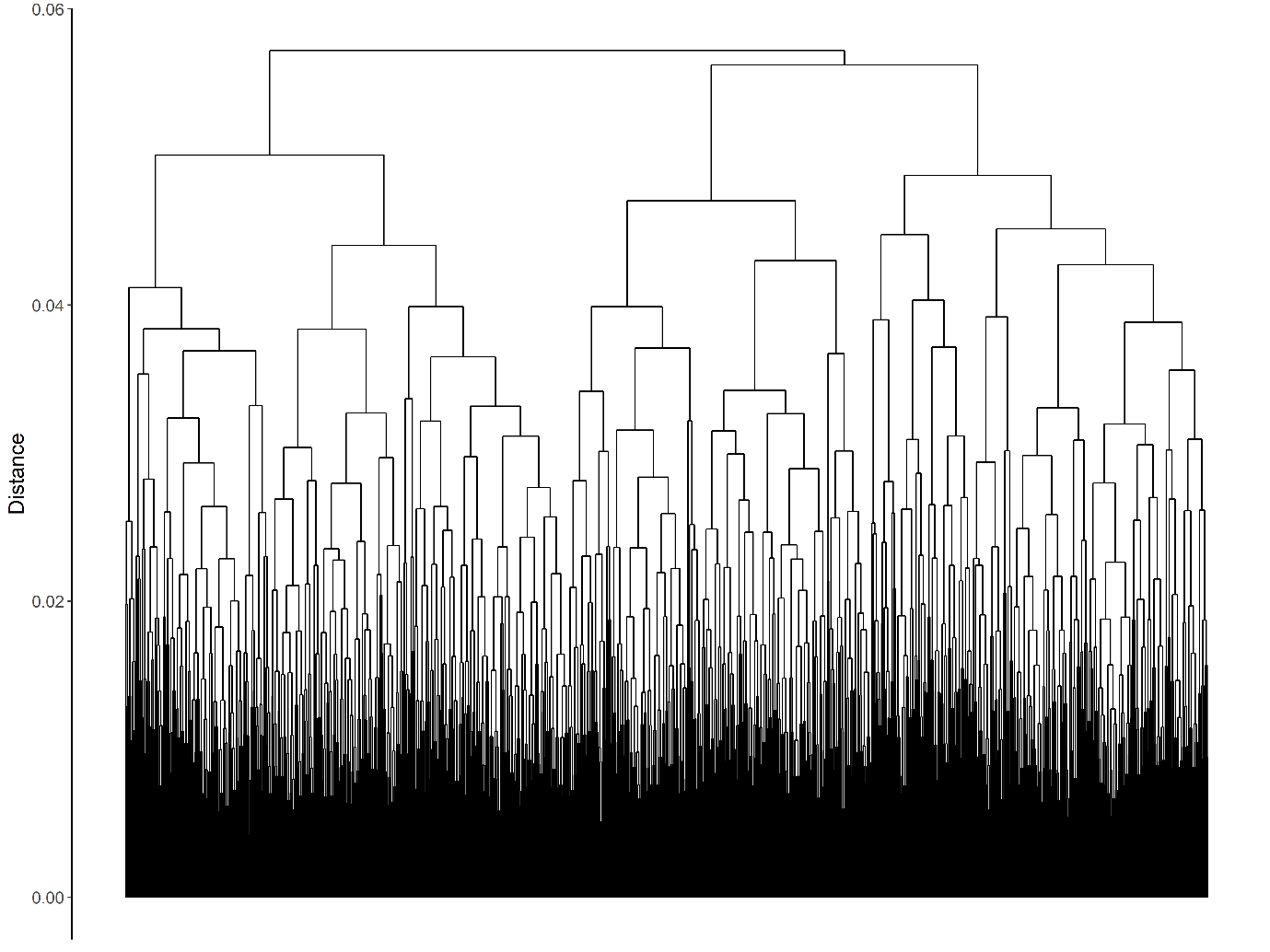


Figure 6 Dendrogram of hierarchical clustering performed on partial correlation matrices of 1000 simulated long time-series (379 years).


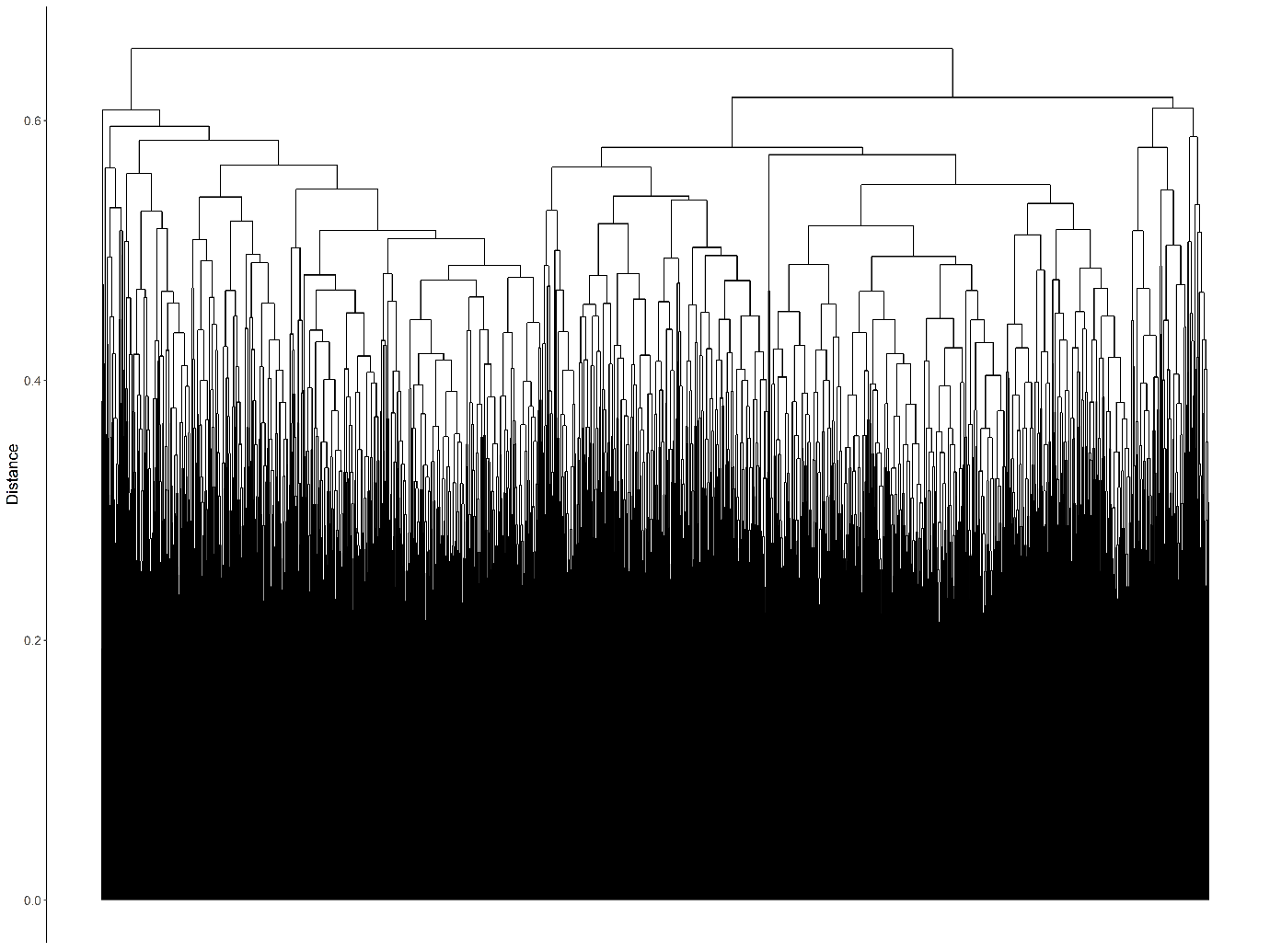


Figure 7 Dendrogram of hierarchical clustering performed on partial correlation matrices of 9000 simulated short time-series (40 years).

We see on the dendrograms that the distances between long time-series were low (the highest distance measured was 0.06) whereas there was more difference between shorter time-series (the highest distance measured was 0.7). We cut the dendrogram on long time-series in four clusters and the dendrogram on short time-series in three clusters.

## Sliding window correlation analysis

We found no evidence for trophic control when estimating correlation between species over longer time-periods (40 and 379 years). The absence of correlation can be explained by two assumptions, either there is really no correlation, or the correlation values are fluctuating over time. We find that the correlation values are fluctuating over time and present the results for the interactions between pelagic fish and demersal and pelagic fish and omnivorous zooplankton in the main manuscript. Here, we provide the graphs from the same analysis, but we present the results for other trophic interactions. We see that fluctuating trophic control is present for all interactions we have explored (Figure 8). The same pattern was found in the reconstructed biomass time-series, even though the variability of trophic control in reconstructed biomass time-series is more limited than the variability of trophic control in the simulated biomass (Figure 9).


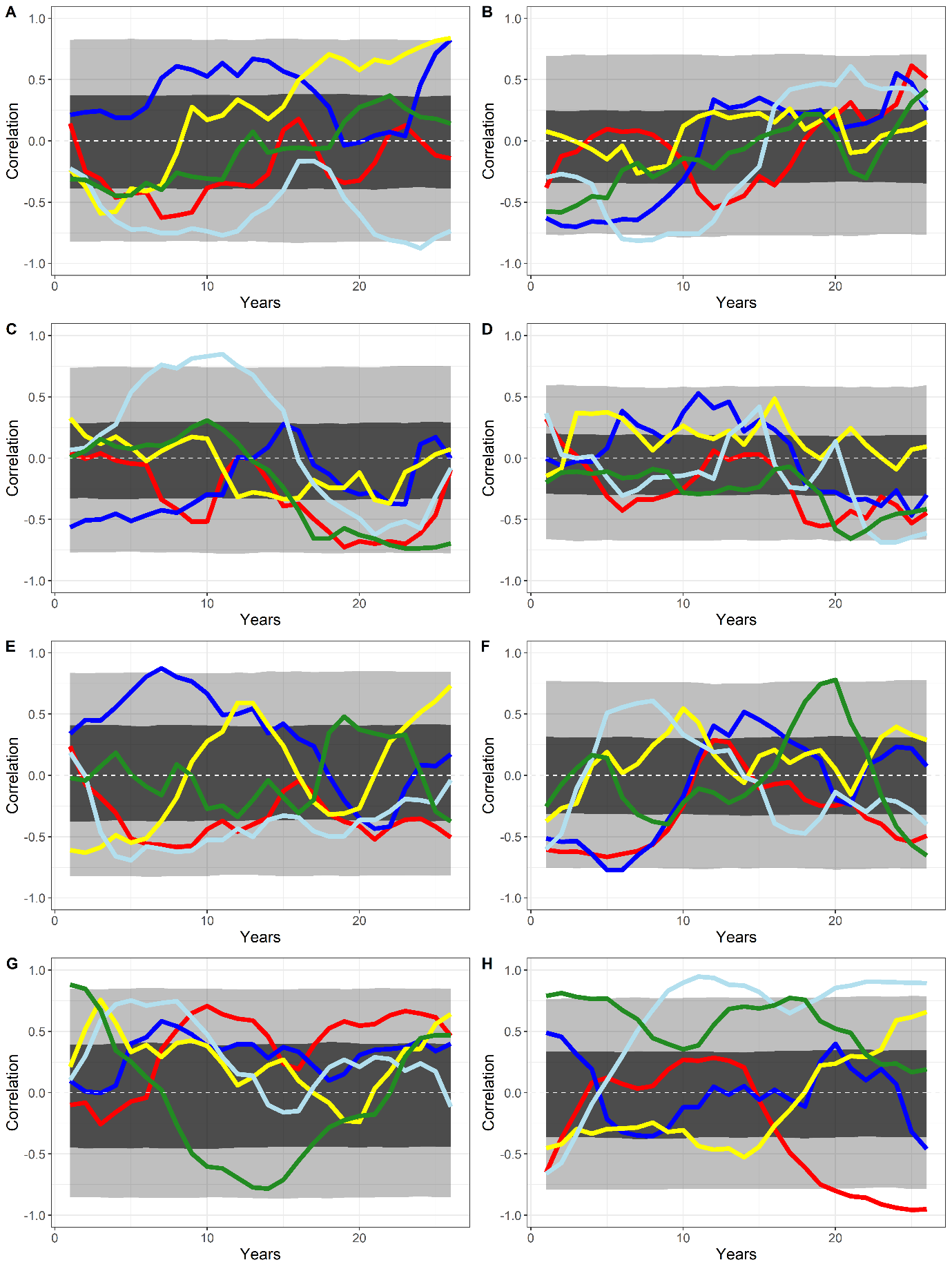


Figure 8 Pearson correlation time series of NDND simulations estimated on 15-years centered sliding window. A: demersal vs. pelagic, B: pelagic vs omnivorous zooplankton, C: demersal vs. omnivorous zooplankton, D: pelagic vs. herbivorous zooplankton, E: mammals vs. pelagic, F: mammals vs. omnivorous zooplankton, G: mammals vs. demersal, H: demersal vs. benthos, colored lines are randomly selected time-series. Grey area is the 95% envelope, black area is the 50% envelope. White dotted line corresponds to a correlation equal to 0.


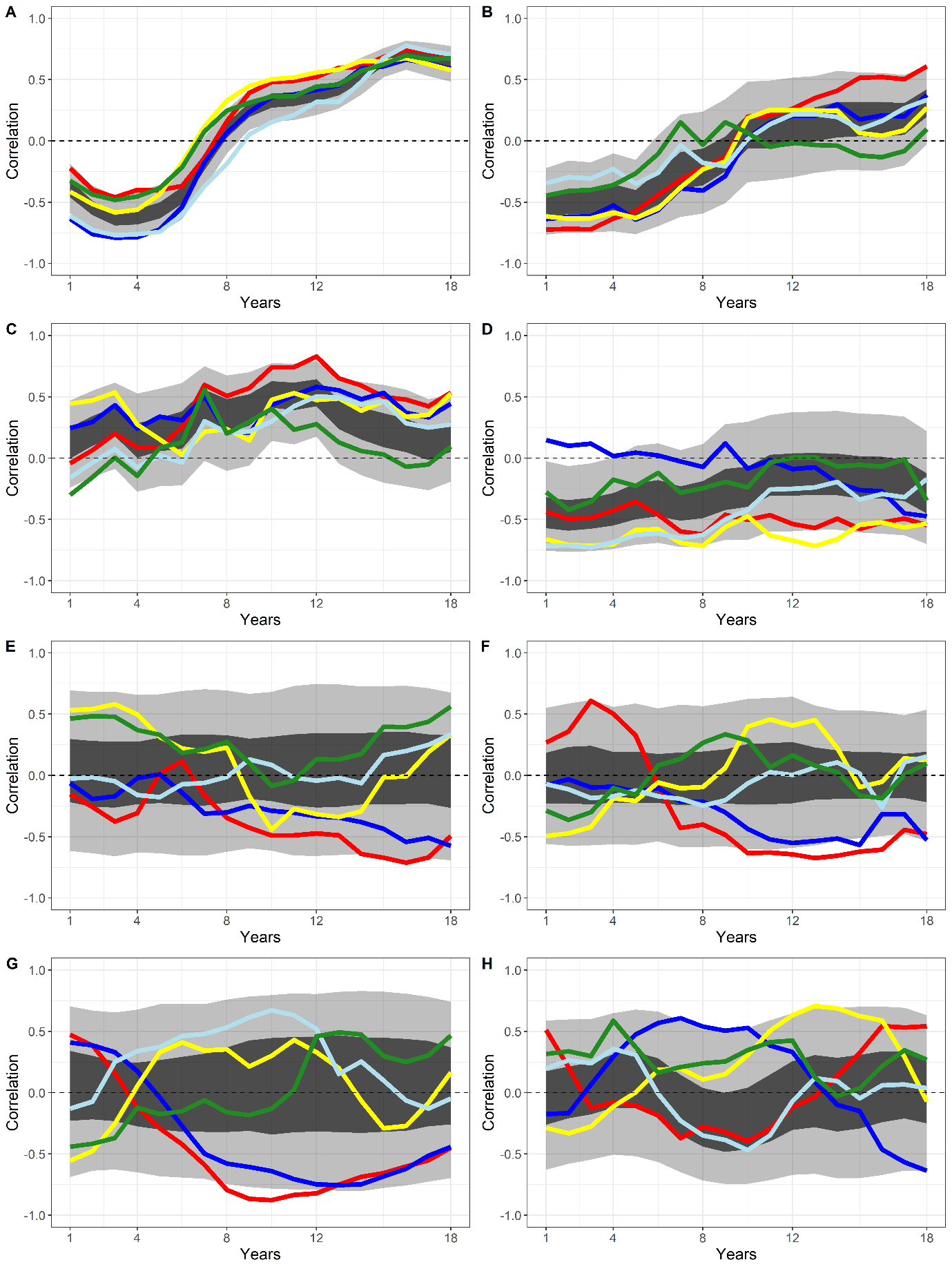


Figure 9 Pearson correlation time series of CaN simulations estimated on 15-years centered sliding window. A: demersal vs. pelagic, B: pelagic vs omnivorous zooplankton, C: demersal vs. omnivorous zooplankton, D: pelagic vs. herbivorous zooplankton, E: mammals vs. pelagic, F: mammals vs. omnivorous zooplankton, G: mammals vs. demersal, H: demersal vs. benthos, colored lines are randomly selected time-series. Grey area is the 95% envelope, black area is the 50% envelope. White dotted line corresponds to a correlation equal to 0.

## Baranov equation

The maximum fished biomass is estimated based on the Baranov equation [6,7]

| $C_{i,t}=\frac{Fmp}{Fmp+M}\left( 1-e^{-\left( Fmp+M \right)} \right)B_{i,t}$ | *(8.1)* |
| --- | --- |

Where *Fmp* is the fishing mortality, *M* is the natural mortality, *C_i,t_* is the catch of species *i* at time *t*, and *B_i,t_* is the biomass of species *i* at time-step *t*. The catch was treated as export (E) in the NDND model.

# References

1. Planque B, Lindstrøm U, Subbey S. Non-Deterministic Modelling of Food-Web Dynamics. Chiaradia A, editor. PLoS ONE. 2014;9: e108243. doi:10.1371/journal.pone.0108243

2. Drouineau H, Planque B, Mullon C. RCaN: a package to fit Chance and Necessity model. 2019.

3. Subbey S, Planque B, Lindstrøm U. Exploring stochasticity and imprecise knowledge based on linear inequality constraints. J Math Biol. 2016;73: 575–595. doi:10.1007/s00285-015-0959-z

4. Planque B, Mullon C. Modelling chance and necessity in natural systems. ICES J Mar Sci. 2020;77: 1573–1588. doi:10.1093/icesjms/fsz173

5. ICES. Working Group on the Integrated Assessments of the Barents Sea (WGIBAR). 2020 [cited 1 Feb 2021]. doi:10.17895/ICES.PUB.5998

6. Baranov FI. On the question of the biological basis of fisheries. Izvestiya. 1918;1: 81–128.

7. Branch TABA. Differences in predicted catch composition between two widely used catch equation formulations. Canadian Journal of Fisheries and Aquatic Sciences. 2009 [cited 13 Oct 2020]. doi:10.1139/F08-196
