# Supplementary material for: Multiple configurations and fluctuating trophic control in the Barents Sea food-web
Source: PLoS One. 2021 Jul 9;16(7):e0254015. doi: 10.1371/journal.pone.0254015 (PMC8270156; doi:10.1371/journal.pone.0254015)
Supplement: Attachment — Submitted filename: Response to reviewers.docx [file pone.0254015.s003.docx]

Editor:

In fact, the original Reviewer 1 still suggests rejection with requests to alter the justification for the study, providing a scientifically justifiable reason for the modeling efforts and constructing a meaningful and useful model that can be used for hypothesis-driven questions.

We understand the concerns raised by reviewer #1. We have revised the introduction to further clarify the purpose of our study, explain why the modelling approach is suitable to achieve this purpose and why the results are of potential interest for the scientific community. We disagree with reviewer #1 about the significance of our research but we would like to remind the editor and reviewer that significance should not be considered since it is not part of the evaluation criteria retained for publication in PlosOne.

Importantly, they also suggest that the aggregation of pelagic species is inappropriate, and I agree.

The aggregation or splitting of species group should be justified by the model purpose. In the present case, we argue that disaggregating pelagic species would not constitute an improvement to address this purpose and that splitting the pelagic fish group will render the model more complex, more difficult to parametrize and the results more difficult to interpret. We present the detailed rationale for not splitting the pelagic trophospecies in smaller groups in the response to reviewer #1.

Reviewer 3 again comments that the mechanisms are not well explored.

The NDND model presented in this study is not designed to explore mechanisms, but to simulate possible food-web dynamics given stochastic trophic flows that lead to biomass changes. The RCaN model is a NDND model constrained by data and thus it has a smaller range of food-web configurations. We argue that some simulated dynamics are similar to the reconstructed dynamics “by chance”. We clarify the mechanism of the NDND and the RCaN models in the response to reviewer #3 as well as in the material and methods section of the revised manuscript.

The manuscript could be greatly improved by considering the reviewer's comments, especially in the introduction and set up for the study. My suggestion is to consider what your model provides, why it is important/unique/novel, and how this as a tool could assist scientists and managers in answering questions moving forward. This would particularly be helpful in justifying your study and set it apart from numerous other modeling efforts.

We have revised the introduction section to emphasize the use of the range of variability of the Barents Sea food-web configurations to define a baseline for the ecosystem variability for management purposes (L 51-53 in the revised manuscript: “*The historical range of variability informs us on how an ecosystem has varied in the past. It can be used as a reference for the variability of an ecosystem to improve the assessment and the prediction of future changes in its dynamics [10].*” ; L 91-93 in the revised manuscript: “*However, there is a need to define a baseline for the variability of the Barents Sea ecosystem to assess if the recent changes in its dynamics may reflect its stochastic variability or if they were induced by variations in anthropogenic drivers*”). As it simulates the possible range of food-web configurations, the NDND model is a suitable tool to assess this baseline.

Reviewer #1:

however, this manuscript still lacks a compelling contribution to the literature.

For instance, the paper says that the Barents Sea is a well studied ecosystem, but then the lack of data is the reason for the study. That rationale does not seem reasonable. It is clear that more data became available after 1985, but even considering that change in data, the previous comment about “well-studied,” is confounded.

The Barents Sea is a well-studied ecosystem compared to many other marine ecosystems. There is a large amount of data available for many species in the Barents Sea, for the last 3-5 decades. This is particularly true for fish and plankton species. Most monitoring programs in the Barents Sea started in the 1980’s. Except for the major commercial species such as Atlantic cod and herring, little is known about dynamics of other components before 1980. Even today, some components of the Barents Sea are poorly sampled and monitored, for example benthos. So, even though the Barents Sea is *relatively* well studied, there are many groups for which data is limited.

To avoid further confusion, we have removed the term “*well-studied*” from the revised manuscript.

In addition, every food web model is considered “one snapshot” of that particular food web, and it is well understood that there are many more possible configurations of a food web from the same system. So, the goal of the manuscript to show how many different configurations of one food web exist, does not provide a meaningful contribution to the system or to food web modeling in general. What would be meaningful, is to develop one food web model, using the best data available, and then asking scientific questions about the system using the model as a tool to answer those questions. In other words, the significance of this contribution still remains questionable.

Although we understand the reviewer’s point, we disagree with it. Reviewer #1 points that “*it is well understood that there are many more possible configurations of a food web from the same system*”. Even if this is true, this does not mean that we know what these other configurations can look like or that we know the range of possible configurations. What we provide in this contribution, using a novel simulation approach, is a quantitative description of possible food-web configurations, including those that have not been observed in the recent past. This is a significant step forward. The results can have direct implications for the design of management policies that need to deal with uncertain future changes.

L 51-53 in the revised manuscript: “*The historical range of variability informs us on how an ecosystem has varied in the past. It can be used as a reference for the variability of an ecosystem to improve the assessment and the prediction of future changes in its dynamics [10].*”

L 91-93 in the revised manuscript: “*However, there is a need to define a baseline for the variability of the Barents Sea ecosystem to assess if the recent changes in its dynamics may reflect its stochastic variability or if they were induced by variations in anthropogenic drivers.*”

The reviewer further suggests that it would be meaningful to “*develop one food web model, using the best data available, and then asking scientific questions about the system using the model as a tool to answer those questions*”. We have done just that. The model is based on decades of data collected in the Barents Sea and elsewhere to structure the food web and set the input parameter values. This model, which is a dynamic-stochastic food web model, is an appropriate tool to answer our question: what could be the range of configurations of the Barents food-web.

L 76-78 in the revised manuscript: “*As such, the NDND model has been designed to reproduce the high variability of natural systems by exploring their “state-space”.*”

Another remaining concern that the authors chose not to address is the combination of pelagic species into one functional group. While many if not all food web models combine species into functional groups, if there are specific aspects and ecological interactions for which the authors are going to consider in the Discussion, then those species should be disaggregated so their trophic responses could be interpreted separately. The response of “resolving trophic interactions at the ecosystem level,” does not provide a substantial rebuttal to the suggestion of disaggregating species for which the authors make individual comments in the Discussion. A trophic interaction, by definition is at the group level, so “ecosystem level trophic interactions,” does not make much sense, unless the authors consider Information Theory and Network Analyses--which provide metrics for ecosystem structure and function.

One should consider splitting functional groups in smaller groups if the aim of the study is to resolve interactions between specific sub-groups or species and the rest of the ecosystem. In other words, if the question of the study does not focus specifically on the trophic interaction between capelin and herring (and the interactions between these two species with other components of the food web), we don’t see the need to further complexify the food-web topology with respect to this specific group. Splitting the pelagic group in smaller groups is not more justified than splitting the demersal fish, benthos, or plankton groups, which are also heterogeneous. By increasing the complexity of the food-web, we might move away from the first purpose of the NDND model, which is to explore the stochastic variability of an ecosystem using a simplified representation of its real food-web.

We have revised the discussion section in the revised manuscript to emphasize the importance of trophic groups in discussion of Barents Sea food webs and removed any reference to species specific interactions.

Reviewer #3:

I appreciate that the authors conducted additional analyses investigating the role of fishing for their result. I´m convinced that fishing was not a driving force of the gained result.

However, I still lack a reasoning as to why one of the simulated biomass configurations is more similar to reconstructed food web configuration. I think the analyses are well done, but I still think that it would be good if the authors delved a bit deeper into the mechanisms leading to the simulated results before publication.

We understand the reviewer’s comment. The NDND model is made of a stochastic and a deterministic part. While the trophic flows are drawn randomly (given that they fulfil the model constraints), the subsequent changes in biomass are fully determined by the model master equation. In return, these new biomasses will set new constraints on the trophic flows in the next time step, and so on. We have edited the material and methods section to emphasize the mechanisms of the NDND model:

L 104-109 in the revised manuscript: “*In the NDND model, the dynamics of the different trophospecies (hereafter named species) result from biomass exchanges between species whose values are sampled randomly (chance), given a set of biological and physical constraints (necessity). Trophic flows define mechanistically the biomass at the next time-step according to the master equation of the model (see supplementary materials S1). Estimated biomass values will then constrain the values of the trophic flows for the next time-step, and so on.*”

The RCaN model used for reconstructing past dynamics relies on the same principles as the NDND model. In addition, RCaN integrates historical data about the species and fluxes to further constrain the dynamics of the food-web. In other words, RCaN is a NDND model constrained by historical data. NDND simulations explore a wider range of possible dynamics and some of these dynamics are similar to the RCaN dynamics “by chance”. We discussed this in two paragraphs in the discussion section in the revised manuscript:

L 420-426 in the revised manuscript: “*The difference in the range of variability between our reconstructions and our simulations ensues from the differences in the constraints between the RCaN model and the NDND model. The RCaN model and the NDND model are based on the same modelling principles: chance and necessity. Yet, the RCaN model is more constrained than the NDND model since it integrates past observations to constrain the food-web trajectories [34]. Thus, we could expect the range of variability of the RCaN reconstructed food-web configurations to be smaller than the range of variability of the NDND simulated food-web configurations.*”

L432 – 438 in the revised manuscript: “*In the NDND model, the dynamics of the Barents Sea food-web is driven by trophic flows [22,23]. At any time-step, the flows are drawn randomly (within the model constraints) and their value determines the species biomass at the next time step. This in turns modifies the constraints and thereby affects the drawing of the trophic flows at the following time step. The overlap between simulated and reconstructed food-web configurations is primarily driven by chance. It indicates that the reconstructed food-web configurations are part of a wider set of configurations that reflect the possible range of stochastic variability of Barents Sea food-web.”*
